# Population Knowledge, Attitude, and Practice Regarding *Helicobacter pylori* Transmission and Outcomes: A Literature Review

**DOI:** 10.3389/fpubh.2017.00144

**Published:** 2017-06-23

**Authors:** Lisa J. Driscoll, Heidi E. Brown, Robin B. Harris, Eyal Oren

**Affiliations:** ^1^College of Medicine, University of Arizona, Tucson, AZ, United States; ^2^Mel and Enid Zuckerman College of Public Health, University of Arizona, Tucson, AZ, United States

**Keywords:** *Helicobacter pylori*, knowledge, attitudes, practices, general population

## Abstract

**Background:**

*Helicobacter pylori* infection is associated with the development of chronic gastritis, peptic ulcer disease, and gastric cancer. Current clinical recommendations are that *H. pylori* test-and-treat should be individualized based on comorbidities and patient preferences among populations at increased risk for certain morbidities. However, knowledge, attitudes and practices regarding *H. pylori* among potential patient populations are largely unknown.

**Materials:**

We conducted a literature review to assess knowledge, attitudes, and practices of patients or community populations around *H. pylori* transmission, prevention, and associated morbidity.

**Results:**

Nine studies met the inclusion criteria, all published between 1997 and 2014. Eight studies evaluated perception of *H. pylori* among at-risk populations, while one study evaluated perception among a general population. The studies suggest inconsistencies between the perceptions of these populations and the established understanding of knowledge, attitude, and preventive practices for *H. pylori* among even at-risk populations.

**Conclusion:**

To adequately respond to current test-and-treat recommendations for treatment of *H. pylori*, general population education must be implemented, especially among at-risk populations. Further work is needed within at-risk populations in the United States to determine prevalence of *H. pylori* and their current knowledge if adequate prevention strategies are to be designed.

## Introduction

*Helicobacter pylori* is a gram-negative bacterium that predominately infects the lining of the stomach. *H. pylori* is associated with the development of chronic gastritis, peptic ulcer disease, and gastric cancer. Worldwide, gastric cancer is the fifth most common cancer and third leading cause of cancer-related death (1). It has been estimated that 78% of all gastric cancer cases, and 89% of non-cardiac cases, can be attributed to chronic *H. pylori* infection (2). The development of gastric cancer from *H. pylori*, which involves a multistep process from chronic gastritis to atrophic gastritis to intestinal metaplasia to dysplasia to gastric cancer, can take decades to develop (3). This slow progression provides an opportunity for early detection and treatment of *H. pylori*, leading to the prevention of gastric cancer.

*Helicobacter pylori s*creening and prevention among at-risk groups may reduce certain diseases and “test-and-treat” should be individualized based on comorbid illness and patient preferences (4). These at-risk populations include individuals with a number of potential indications, including a confirmed history of peptic ulcer disease and gastric MALT lymphoma (4). In the United States, higher prevalence of *H. pylori* has been reported among individuals living close the US/Mexico border, as well as among American Indians and Alaska natives (5–7). However, among at-risk populations, the current level of knowledge and behaviors and risk perception are unknown. This information is needed if “test-and-treat” strategies are to be successfully employed for specific populations. The aim of this review is to examine the current literature with respect to public perceptions of the impact of *H. pylori*.

## Materials and Methods

A framework to assess knowledge, attitudes, and practices (KAPs) was defined based on the World Health Organization *Guide to Developing Knowledge, Attitude, and Practice Surveys* (8). In the context of this review, “Knowledge” referred to general knowledge about *H. pylori* including transmission, course of infection, disease sequelae, risk/protective factors, diagnosis, treatment, and prevention. “Attitudes” referred to individual, peer, and community risk perception of getting *H. pylori* or its disease sequelae. “Practices” corresponded to actions people might take to prevent *H. pylori* infection, including hand washing, safe food preparation, and source of drinking water. These latter practices were sought because evidence suggests fecal–oral transmission may play a role in *H. pylori* transmission (9), and these practices were outlined by the National Institutes of Health as ways to reduce chances of *H. pylori* infection (10).

A literature search was conducted in April 2017 in PubMed using (“*Helicobacter”* OR *“H. pylori”*) AND (“survey” OR “questionnaire”) AND (“knowledge” OR “attitudes” OR “practices”) as search terms. Inclusion criteria were that the study collected primary data from at-risk or members of the general population and that the study incorporated a survey to assess participants own knowledge, attitudes, or practices regarding *H. pylori*. There were no other exclusion terms (e.g., language or date of publication).

## Results

The initial search terms yielded 133 results, with two additional studies identified through follow-up of references. After title and abstract screening to exclude articles not focused on potential patient KAP, 15 articles remained. After reviewing full text, an additional six studies were rejected because the survey included only demographic and/or clinical history without any assessment of KAP (*n* = 5), or *H. pylori* was not the primary focus of study (*n* = 1).

Of the nine studies included in this review, two were conducted in eastern China (11, 12), two in South Korea (13, 14), two in North America (15, 16), and one each in Ethiopia (17), India (18), and Malaysia (19). All studies focused on populations at increased risk for gastric cancer, except the US population-based survey to assess and promote awareness (15). Within this search, no publications were found which specifically addressed gaps in KAP among the general population regarding risks associated with acquiring *H. pylori* infection. All studies were published between 1997 and 2014. For each study, the type of questions used in the survey tool were classified into demographic, clinical history, knowledge, attitude, or practice related questions. A summary of the questions (along with results) is presented in Table 1.

**Table 1:** Summary of questions and replies nested within studies and ordered by publication date for knowledge, attitudes, and practices. Word-for-word questions taken from literature are indicated by quotes.

Knowledge						
Study	Question	Results				
Wynne et al. (16)	Asked their opinion on how people get HP	Mention water: 26%			Do not mention water: 74%	
	
Shin et al. (14)	*(9 statements read to respondent; respondent then asked to answer yes, no, or don’t know)*	**Yes**		**No**	**DK**	**Missing**
	
	“More than 50% of Korean adults have HP in their stomach”	45.2%		4.1%	45.9%	4.8%
	“Transmission of the bacteria usually occurs through mouth among family members”	31.1%		12.6%	49.5%	6.8%
	“HP infection often disappears spontaneously”	19%		28.6%	45%	7.3%
	“HP is known to cause gastric cancer”	58.3%		4.8%	32.9%	4%
	“HP can cause gastric or duodenal ulcer”	60.8%		2.2%	32%	5.1%
	“HP does not cause gnawing pain or dyspepsia”	15.2%		37.2%	40.9%	6.6%
	“HP can be identified by taking small pieces of tissue from the stomach during endoscopy”	50.8%		8.4%	35.1%	5.6%
	“HP can be treated by drinking yogurt”	17.1%		34.3%	42.7%	36%
	“There is effective treatment for HP”	45.5%		5.6%	42.7%	6.1%
	
Xia et al. (12)		**HP positive**			**HP negative**	
	
	“Have you heard of *Helicobacter pylori*?”	Yes: 22%		No: 78%	Yes: 35%	No: 65%
	
	“In your opinion, how is *Helicobacter pylori* transmitted?” *(read answer choices)*					
	“Eating unclean food”	27%			42%	
	“Sharing utensils/chopsticks”	26%			35%	
	“Bodily fluids from infected person”	13%			12%	
	“Don’t know”	34%			11%	

Oh et al. (13)	“How much do you think the following factors influence the risk of developing gastric cancer?” *(0–100%; 0 means no association, 100% means certain association)* Age, sex, family history of gastric cancer, salty diet, diet of charred foods, spicy diet, processed ham or sausages, fatty or greasy diet, vegetables or fruits, bacteria (e.g., HP), alcohol, smoking, gastric ulcer, chronic gastritis, previous gastrectomy history, stress, obesity, physical inactivity, air pollution	Stress 73.5% Chronic gastritis 72.1% Gastric ulcer 71.2% Previous gastrectomy 68.7% Charred foods 67.3%				
	“How much do you think that gastric cancer can be prevented when the following factors are totally removed?” *(0–100%; % of gastric cancers that could be prevented if risk factor removed)* Age, sex, family history of gastric cancer, salty diet, diet of charred foods, spicy diet, processed ham or sausages, fatty or greasy diet, vegetables or fruits, bacteria (e.g., HP), alcohol, smoking, gastric ulcer, chronic gastritis, previous gastrectomy history, stress, obesity, physical inactivity, air pollution	Stress 68.9% Vegetables or fruits 67% Chronic gastritis 66.7% Gastric ulcer 66% Previous gastrectomy 65.1%				
	“What percentage of gastric cancer do you think is genetically predetermined?” *(write in%)*	38.5%				
	“What percentage of gastric cancer do you think is preventable by modification of lifestyle?” *(write in%)*	60.4%				
	“What is the usefulness of regular screening for the early detection of gastric cancer?”	Very helpful: 61.4%		Helpful 37.4%	Not helpful: 1%	
	“What percentage of gastric cancer do you think is preventable by regular screening?”	Most (54%) said 80–99%				

Chen et al. (11)	“Knowledge rate about HP”	33.2%				
	“Knowledge of transmission route of HP”	23.8%				

CDC (15)	“Stress causes ulcers. Do you agree or disagree?”	Agree: 60%; assoc. w/18–24y, income <$15,000				
	“Eating spicy foods causes ulcers. Do you agree or disagree?”	Agree: 17%; assoc. w/18–24y, income <$15,000				
	“Bacterial infection causes ulcers. Do you agree or disagree?”	Agree: 27%; assoc. w/increased age				
**Attitudes**						
**Study**	**Question**	**Results**				

Shin et al. (14)	Risk perception of gastric cancer compared with people of same age and gender.	Much lower: 14.9%	Lower: 25.5%	Same: 32.3%	Higher: 19%	Much higher: 1.3%
	
Xia et al. (12)	“Do you think you could be infected with *Helicobacter pylori*?”	Yes: 14%		No: 86%		
	
Oh et al. (13)	“What do you think of your self-risk of developing gastric cancer?”	Very low: 9.7%	Low: 36.3%	Average: 40.1%	High: 9.8%	Very high: 1.2%
**Practices**						
**Study**	**Question**	**Results**				

Abebaw et al. (17)	Hand washing after using toilet	No association with HP				
	Hand washing before meal	Positive association with HP				
	Drinking water source	“unprotected surface” has positive association with HP “piped tap” has no association with HP				

Wynne et al. (16)	In past 12 months, consumed water that was not treated at a water Tx treatment	Yes: 40%				
	If yes to above, consumed water directly from river/lake	Yes: 34%				
	In lifetime, consumed untreated water	Yes: 73%				

Xia et al. (12)	Assessment of dietetic hygiene	Better dietetic hygiene has negative association with HP				

Lee et al. (19)	Source of drinking water	“well water” has positive association with HP “tap water” has no association with HP				
	Frequency of boiling water	No association with HP				
	Hand washing after toilet	“not always” has positive association with HP				
	Hand washing before meal	“not always” has positive association with HP				
	Using fingers to consume food	No association with HP				

Oh et al. (13)	“Do you receive regular screening for gastric cancer?”	Yes: 54.2%		No: 45.8%	
	“If not, why do you choose not to undergo regular screening?” *(write in answer)*	No symptoms: 61.8% Busy: 12.6% Fear of detection of cancer: 7.2% Economic problem: 6.6% Concern of process of gastroscopy: 6.4% No info about screening: 2.8% No effect of screening: 1.3% Other: 1.3%				

Ahmed et al. (18)	CWI, calculated based on answers to following questions: Store/reuse water (↑ to ↓ index: never, sometimes, always) Frequency of bathing (↑ to ↓ index: >2 per wk, 1–2 per wk, ≤1 per wk) Boiling water before drinking (↑ to ↓ index: consistent, sometimes, never)	High CWI: 33.3% HP+		Medium CWI: 80% HP+	Low CWI: 88.2% HP+	
	Source of drinking water	“well water or river” has positive association with HP				

Chen et al. (11)	Eating raw vegetables and fruits	Positive association with HP infection				
	Never washing raw vegetables and fruits before eating	Positive association with HP infection				

*Word-for-word questions taken from literature are indicated by quotes*.

*HP, Helicobacter pylori; DK: do not know; CWI, Clean water index*.

### Knowledge

Six studies reported on *H. pylori* knowledge (11–16). General knowledge about *H. pylori* was poor across all studies. Of the two studies that asked whether participants had heard of *H. pylori*, only 22–35% of respondents answered “yes” (11, 12). Interestingly, one study found that those who tested negative for *H. pylori* had heard of *H. pylori* more often than those who tested positive (12).

Knowledge about *H. pylori* transmission was also generally poor. When asked how people can acquire the infection, only 26% of participants mentioned water (16). When asked whether oral transmission was usual among family members, 31.1% of participants responded “yes,” 12.6% responded “no,” and 49.5% responded “do not know” (14). When asked about transmission, people who tested positive for *H. pylori* showed less understanding than those who tested negative (12). One study, asking about transmission found only 23.8% of respondents correctly answered that *H. pylori* can be transmitted by unsafe food preparation and water sources (11).

The two studies from South Korea evaluated knowledge of *H. pylori* as it relates to gastric cancer. Among healthy Korean adults undergoing screening *via* upper endoscopy 58.3% of respondents answered “yes,” that *H. pylori* is known to cause gastric cancer, while 4.8% answered “no,” and 32.9% responded “do not know” (14). Randomly selected respondents in the South Korean population-based survey were asked to what degree individual factors were associated with developing and preventing gastric cancer (13). Respondents believed stress was the highest risk factor for developing gastric cancer, followed by gastric lesions (including chronic gastritis, gastric ulcer, and previous gastrectomy) and eating charred foods. Respondents believed that removing stress would have the greatest impact on preventing gastric cancer, followed by eating vegetables and fruits, and removing gastric lesions.

Two studies evaluated knowledge of *H. pylori* and its relationship to peptic ulcer disease. Shin et al. (14) asked if *H. pylori* can cause gastric or duodenal ulcers; 60.8% answered “yes,” 2.2% answered “no,” and 32% answered “do not know” (14). A US population-based survey from 1997 asked respondents whether stress causes ulcers (yes: 60%), eating spicy foods causes ulcers (yes: 17%), and bacterial infection causes ulcers (yes: 27%) (15).

### Attitudes

Three studies asked questions regarding attitudes related to *H. pylori*/gastric cancer (12–14). All three of these studies assessed perception of self-risk for either contracting *H. pylori* or developing gastric cancer. Shin et al. (14) found that most people viewed their own risk as “same” or “lower” when compared with people of same age and gender. Oh et al. (13) found that most people viewed their own risk for developing gastric cancer as average or low. Xia et al. (12) found that 86% of people did not think they were infected with *H. pylori* despite that within this population, *H. pylori* prevalence was 41%.

### Practices

Seven studies asked questions related to *H. pylori* prevention practices (11–13, 16–19). Generally, good hand washing practices after using the toilet and before eating/preparing meal, safe food practices, and drinking water from a clean source were associated with less *H. pylori* infection. Interestingly, Abebaw et al. (17) found hand washing before meals was associated with higher prevalence of *H. pylori*, and hand washing after using toilet carried no association with *H. pylori* prevalence. However, this result conflicts with what is generally understood about this association, and other studies reviewed here found that hand washing “not always” after toilet and “not always” before meal were associated with higher *H. pylori* prevalence (19).

## Discussion

This review identified nine studies that surveyed public KAPs regarding *H. pylori*. The limited number of publications on this topic is surprising given the potential benefit of *H. pylori* “test-and-treat” in preventing gastric cancer as well as the need for involvement of patient preferences in testing and treatment decisions for at-risk populations (4). The lack of studies in the US is disconcerting given that, while trends in gastric cancer mortality from 2004 to 2013 are decreasing nationally among all other ethnicities, they remain unchanged among American Indian/Alaska Natives (SEER.cancer.gov) (20).

Based on this review, there appears to be limited knowledge about *H. pylori* among the general population, especially with respect to transmission and its association with gastric cancer. Interestingly, the association between *H. pylori* and ulcers may be more widely known than the association between *H. pylori* and gastric cancer (14). However, it appears that many people regard stress as the highest risk factor not only for ulcers, but for gastric cancer as well (13, 15).

This literature review highlights lack of studies evaluating public awareness of *H. pylori*, particularly among populations at increased risk for gastric cancer. With this insight, provider- and patient-based strategies such as screening, surveillance, and outreach programs can be developed to reduce gastric cancer attributable to *H. pylori* infection among these at-risk subpopulations (21, 22).

## Author Contributions

LD and EO developed the original scope of the review. LD and HB conducted the literature search and review. All authors were involved in manuscript preparation, approved the final version, and agreed to be accountable for all aspects of the work.

## Conflict of Interest Statement

The authors declare that the research was conducted in the absence of any commercial or financial relationships that could be construed as a potential conflict of interest.
